# The Underestimated Prevalence of Neglected Chronic Pelvic Pain in Women, a Nationwide Cross-Sectional Study in France

**DOI:** 10.3390/jcm10112481

**Published:** 2021-06-03

**Authors:** François Margueritte, Xavier Fritel, Marie Zins, Marcel Goldberg, Henri Panjo, Arnaud Fauconnier, Virginie Ringa

**Affiliations:** 1 Centre for Research in Epidemiology and Population Health, INSERM U1018, Gender, Health Sexuality Team, 16 Avenue Paul Vaillant Couturier, 94800 Villejuif, France; xavier.fritel@univ-poitiers.fr (X.F.); henri.panjo@inserm.fr (H.P.); virginie.ringa@inserm.fr (V.R.); 2 INSERM CIC 1402, Université de Poitiers, CHU de Poitiers, 86000 Poitiers, France; 3 Population-Based Epidemiological Cohorts-INSERM, Paris Saclay University, UVSQ, 94800 Villejuif, France; marie.zins@inserm.fr (M.Z.); marcel.goldberg@inserm.fr (M.G.); 4 Department of Gynaecology and Obstetrics, Intercommunal Hospital Center of Poissy-Saint-Germain-en-Laye, 10 Rue du Champ Gaillard, 78103 Poissy, France; arnaud.fauconnier@ght-yvelinesnord.fr; 5 Population-Based Epidemiological Cohorts-INSERM, Université de Paris, 75006 Paris, France; 6 Research Unit 7285, Risk and Safety in Clinical Medicine for Women and Perinatal Health, Paris-Saclay University, UVSQ, 78180 Montigny-le-Bretonneux, France

**Keywords:** women’s health, dysmenorrhea, dyspareunia, chronic pelvic pain, non-menstrual chronic pelvic pain

## Abstract

Dysmenorrhoea, dyspareunia, and non-menstrual chronic pelvic pain (NMCPP) are symptoms that are probably underreported and neglected. This study aimed to assess the prevalence and overlapping relations between these symptoms among a general population of French women of reproductive age. A cross-sectional study among the nationwide CONSTANCES cohort study recruiting a representative sample of women within different French areas was constructed. Women aged 18–49 years (*n* = 21,287) who reported periods in the previous three months and experienced intercourse at least once were asked about prevalence of three types of chronic pelvic pain: mild, moderate and severe dysmenorrhea; dyspareunia assessed according to its frequency; NMCPP from a binary question. Between the start of 2012 through the end of 2017, 21,287 women were enrolled, 39.8% of them (95% confidence interval (CI), 39.2–40.5) reported moderate to severe dysmenorrhea; 20.3% (95% CI, 18.7–21.9) of the youngest group (18–24 years) reported severe dysmenorrhea. Dyspareunia was reported to happen often or always by 7.9% (95% CI, 7.5–8.2) and peaked among the youngest women at 12.8% (95% CI, 11.5–14.1). NMCPP was reported by 17.0% (95% CI, 16.5–17.5). Moreover, 7.5% (95% CI, 6.4–8.6) of the women reported two or more types of severe or frequent pain. More attention should be paid to this substantial proportion (7.5%) of French women of reproductive age who experience multiple, severe and frequent pelvic pain symptoms.

## 1. Introduction

Chronic pelvic pain symptoms among women of reproductive age are one of their most frequent reason for seeking health care [1,2]. These symptoms generally include at least one of three types of pain: dysmenorrhea, dyspareunia, and non-menstrual chronic pelvic pain (NMCPP, not related to period or intercourse) [3,4].

Their prevalence among the general population are currently unknown, because of various methodological issues (small samples or from other studies not designed for this purpose) and bias when collecting data (estimates by telephone interviews or postal surveys). Moreover, women barely seek medical care for menstrual symptoms because they believe them to be part of the “female condition” especially among young people whereas pain acceptance among dysmenorrhea appears to be associated with better physical and mental quality of life [5,6]. Thus, this situation may also explain the failure to complain about dyspareunia or NMCPP among women [7,8]. This observation may reflect an attitude in the general population that prevents women from seeking and doctors from providing the appropriate clinical care [3,9,10,11]. It may also reveal that chronic pelvic pain is an underestimated and neglected symptom that women have to deal with. 

Accordingly, using a health system database appears unreliable for estimating the prevalence of chronic pelvic pain symptoms because it represents only people that are using this health care system. Those who do not seek medical care for many reasons are not referenced in these databases although they may have symptoms that potentially require medical advice. Also, a cohort specially designed for chronic pelvic pain among women is exposed to the risk of selection bias: women who agreed to participate were more likely to have chronic pain because they could be believed to have a better follow-up. Then, to have a reliable estimation for this purpose, a cohort draw from the general population and designed for many other topics than chronic pelvic pain seems a better alternative.

No general population-based data describe the current burden of chronic pelvic pain among women in France. Abroad, several studies have collected data but most of the time from small general population samples, like Pitts et al. with a sample of almost 2000 women in Australia [10]. On the other hand, other studies were constructed using healthcare data which were not designed for this purpose like Zondervan et al. [11]. Then, having a consistent sample drawn from a general population questioned not only for chronic pelvic pain (CPP) but also for other medical or epidemiological issues seems to fit better to the purpose. However, the population in France is quite different from other European or northern American countries because of differences within health care systems or socio-economic inequalities, leading to the impossibility of having any reliable estimates on CPP in France on the basis of previous studies abroad [12].

The CONSTANCES cohort study, designed for several epidemiological purposes, provides a new French database of information collected from the general population of France. This nationwide cohort fits our objective of estimating the prevalence of dysmenorrhea, dyspareunia, and NMCPP among women in the general population, according to their severity and overlapping relations [13,14].

We further hypothesize that these three types of chronic pelvic pain are highly prevalent symptoms and that their prevalence may be different according to age. The possibility that women may present more than one of these symptoms at the same time underlines the importance of examining their interrelations.

## 2. Material and Methods

### 2.1. Design of the CONSTANCES Cohort Project

The CONSTANCES cohort is a “general purpose” population-based epidemiological cohort comprising a randomly selected sample of 200,000 French adults aged 18–69 years at inclusion and affiliated with the General Health Insurance Fund (about 85% of the French population not including self-employed and agricultural workers) in different regions across the country [13,14,15]. This cohort was specially designed to recruit people from the general population and to collect data about numerous topics (quality of life, income, work, women’s health, respiratory health, visual health, musculo-skeletal disorder) that could lead to many epidemiological studies. People who are likely to be invited should live in an area of France where a health screening centre (located in different areas across the country), was available and affiliated to the CONSTANCES cohort project. Women included within the CONSTANCES cohort were offered a free medical examination and asked to complete a variety of surveys (lifestyle, quality of life, reproductive and gynaecological health, and medical history) at enrollment. Recruitment began in late 2012 and was still ongoing at the time of the study. People recruited to be part of this project were randomly sent a postal invitation (based on the number of the General Health Insurance Fund). Algorithms and statistical methods to invite people being part of this project were used to avoid selection bias, as the participation rate after the first postal invitation to be part of the cohort was estimated at 7% [13].

In order to minimize selection effects on estimates calculated from data cohort, “a control cohort”, drawn from a random sample of non-participants, was designed with 400,000 non-participants who were previously invited to be part of the cohort but declined [14]. This “control cohort” was made of data from the General Health Insurance Fund. This sample was not designed to be used for studies but only to build sampling weights for every people included in the cohort that are calculated a few years after inclusion. When using the reweighting techniques previously described, we were able to calculate more reliable prevalence taking into account potential biases such as selection biases [16].

### 2.2. Study Design

Women included for this study were all those aged 18–49 years within the CONSTANCES cohort from the beginning of the recruitment (late 2012) until the end of 2017. We used only data at inclusion and no follow-up data as the recruitment was still ongoing when the analysis was performed. Each woman who agreed to participate was asked on enrollment in CONSTANCES to complete a self-administered questionnaire, comprising questions previously developed in France in order to evaluate endometriosis-related pain symptoms [17,18]. These items assess the intensity or frequency of dysmenorrhea, dyspareunia, and NMCPP [4]. The topic of chronic pelvic pain was investigated only in women younger than 50 years given that the proportion of perimenopausal and post-menopausal women rises continuously from this age. We selected women who reported menstrual periods during the three months before inclusion and who had already experienced intercourse. Women who did not have periods in the last three months due to a specific treatment (contraceptive pill, hormonal intrauterine device) or condition (breastfeeding…) or for other causes (surgery such as bilateral salpingo-oophorectomy or hysterectomy) were not asked to answer about dysmenorrhea and NMCPP and were not included in this study. Those who answered about dysmenorrhea where they did not have to, because they did not have periods in the last months, were not included in this study.

For dysmenorrhea, whether or not primary or secondary, women were asked to rate the severity of pain during their previous three periods on a numeric pain scale from 0 (no pain) to 10 (worst pain) without any example or commentary about thresholds (the question was *How do you assess the average intensity of your pain during period? Check the box that best fits your situation* with each box for a number from 0 to 10). This numeric rating scale, is a scale advised to assess dysmenorrhea or other pelvic painful symptoms [19,20]. For this study, we considered that pain rated 0 was considered “no pain”, between 1 and 3 “mild”, between 4 to 6 “moderate”, and 7 or higher “severe»; as a reduction of about 30% of the score in a numeric rating scale reflect a moderate clinically important change according to Dworkin et al. [21]. For dyspareunia, pain during or immediately after intercourse, (the question was *Do you ever have pain during intercourse*, or immediately afterwards?), women could choose as answers: none or rare, sometimes, often, always, or I would prefer not to answer; no time period for evaluating symptoms was specified. Women reporting sometimes, often, and always dyspareunia were considered to experience it. Women who preferred not to answer were excluded. Those answering often and always dyspareunia were reported together. For NMCPP, women were also asked whether they regularly experienced (*yes or no*) other types of chronic pelvic pain, not during periods, without any specification of a time period (the question was *Apart from pain period, do you regularly have pain localized in lower abdomen?*).

### 2.3. Statistical Analysis

Baseline characteristics of women are presented per age group (in 5-year ranges, except for the youngest). We assessed the prevalence of the three types of chronic pelvic pain in the overall sample and then per age group with their 95% confidence intervals (CI) using the Wald estimator. We also performed regression linear trend tests for every estimated pain prevalence to assess age-related variation. A proportional Venn diagram was used to illustrate relations between these three types of pain.

Sampling weights are calculated for each year from people invited to participate in the cohort according to a stratified sampling scheme [14,22]. Then each participant to the cohort CONSTANCES will be attributed a weight, calculated from the non-participant cohort, that may prevent from potential selection biases when estimating the prevalence of chronic pelvic pain. As of now, weights have been calculated only for the inclusions in 2013 and 2014. Then, to assess the reliability of our findings, we performed a sensitivity analysis comparing the prevalence of each type of CPP for each age class with or without weighting techniques only for the years available. STATA© 15.1 IC (StataCorp, College Station, TX, USA) was used for the analysis.

## 3. Results

Between late 2012 through the end of 2017, the CONSTANCES cohort enrolled 63,430 women and from them, 34,498 were aged 18–49 years. For the 26,720 women having periods in the last three months, 24,633 answered question about dysmenorrhea, 24,763 about NMCPP. For those who had experienced intercourse once, only 30,852 answered about dyspareunia. As we selected only women who answered the three questions, this analysis considers the 21,287 women aged 18–49 years. The distribution of women in each age group varied from 2386 for the youngest to 4180 for the 40–44 year olds. Baseline characteristics among age groups (body mass index, smoking status, employment status, geographic origin, and contraceptive method) are summarized in Table 1.

**Figure 1:**
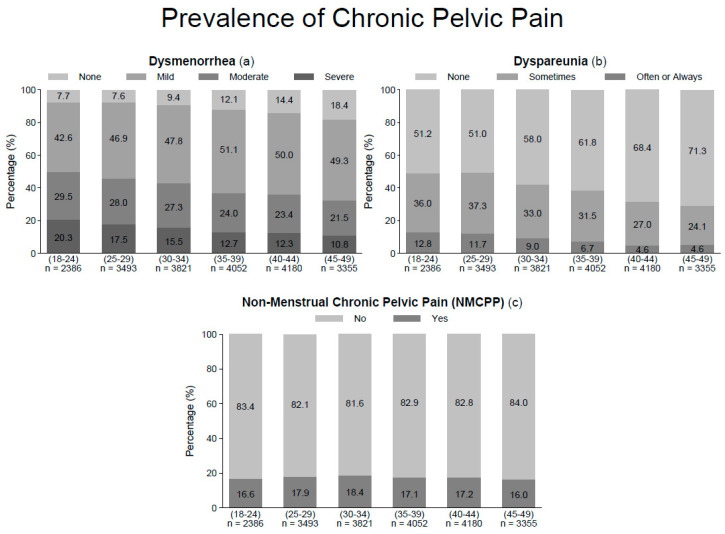
Prevalence of each type of chronic pelvic pain, *n* = 21,287. (**a**) Prevalence of dysmenorrhea (none/mild/moderate/severe), (**b**) Prevalence of dyspareunia (none/sometimes/often or always), (**c**) Prevalence of non-menstrual chronic pelvic pain (no/yes).

Prevalence of moderate to severe dysmenorrhea, dyspareunia (sometimes, often or always) and NMCPP was, respectively, 39.8% (95% confidence interval (CI), 39.2–40.5), 39.0% (95% CI, 38.4–39.7) and 17.3% (95% CI, 16.8–17.8) among the 21,287 women selected. This prevalence peaked among the youngest women (18–24 years old) and was lowest among the oldest (45–49 years old), i.e., severe dysmenorrhea decreased from 20.3% (95% CI, 18.7–21.9) in the youngest group to 10.8% (95% CI, 9.7–11.9) in the oldest, and moderate dysmenorrhea from 29.5% (95% CI, 27.7–31.3) to 21.5% (95% CI, 20.1–22.9) respectively, with a significant linear trend (*p* < 0.001) for both. Inversely, the prevalence rose in the oldest women, from 42.6% (95% CI, 40.6–44.6) to 49.3% (95% CI, 47.6–51.0) for mild dysmenorrhea and that of no dysmenorrhea from 7.7% (95% CI, 6.6–8.8) to 18.4% (95% CI, 17.1–19.7) respectively, also with a significant linear trend (*p <* 0.001, Figure 1).

A similar linear trend was found between dyspareunia and age. Prevalence of pain during intercourse happening often or always was 7.9% (95% CI, 7.5–8.2) and peaked among the youngest group at 12.8% (95% CI, 11.5–14.1). It decreased among older women, with a nadir of 4.6% (95% CI, 3.9–5.3) among the oldest group, again with a significant linear trend (*p* > 0.001). The same was observed among the women reporting occasional dyspareunia and those with no or rare dyspareunia increased significantly for the oldest from 51.2% (95% CI, 49.2–53.2) to 71.3% (95% CI, 69.8–72.8), for the youngest (*p <* 0.001, Figure 1).

The prevalence of NMCPP was estimated at 17.0% (95% CI, 16.5–17.5). It increased up to the 30–34 age group and decreased in the oldest group with a significant U-shape trend (*p* < 0.001, Figure 1).

**Figure 2:**
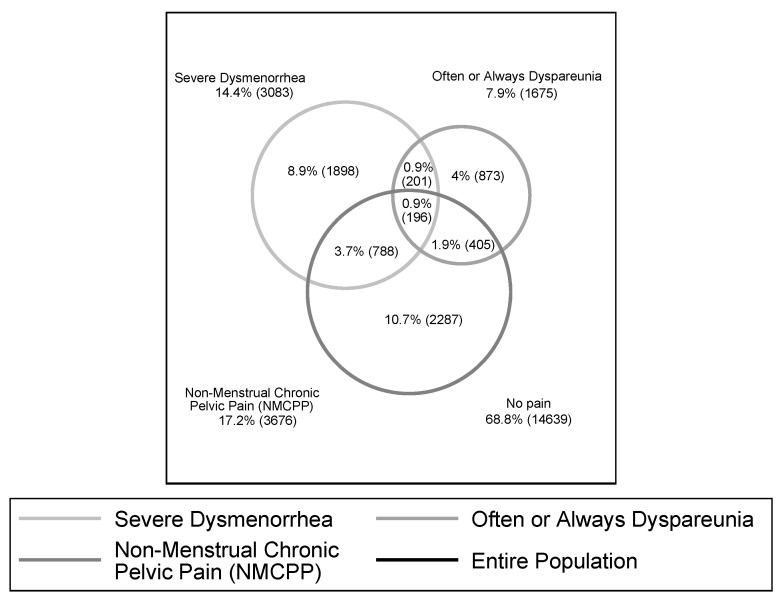
Proportional Venn diagram drawing overlapping relations between symptoms of the three types of chronic pelvic pain according to its severity (dysmenorrhea) or frequency (dyspareunia), *n* = 21,287.

Because women could have reported more than one type of chronic pelvic pain, we assessed the overlap between severe dysmenorrhea, often or always dyspareunia, and NMCPP using a proportional Venn diagram (Figure 2). Almost a third of women experienced at least one severe or frequent form of chronic pelvic pain, and 1587 women (7.5%, 95% CI, 7.1–7.9), (Figure 2) reported two or three severe or frequent symptoms.

Sampling weights were only available for each participant included in 2013 and 2014. Sensitivity analysis about the prevalence of the three components of CPP (none, mild moderate and severe dysmenorrhea/none, sometimes often or always dyspareunia/NMCPP) for each age group concerned only 6191 women. Differences between prevalence rates with and without weights were minimal within each age group and type, with a mean difference of −1.9 × 10^−7^% and a standard deviation of 1.7% (Figure S1). The strongest variations between prevalence calculated with or without weights (maximum almost 5%) appeared when prevalence was high (Figure S2). On the contrary, when a prevalence rate was low, the variation in estimating prevalence with or without reweighting was minimal (slightly above 1%). Furthermore, the mean for misspecification effects (assessing bias in variance estimators) was estimated at 1.5 and varied from 1.3 to 1.7; this means that the estimation bias was minimal among the women included in 2013 and 2014.

## 4. Discussion

### 4.1. Main Findings

In our sample, young women were more likely than their older counterparts to report severe dysmenorrhea. A similar linear age relation was observed for the frequency of dyspareunia. The prevalence of NMCPP increased until it reached its highest level among women aged 30–34 years and then decreased. Overall, our finding that 7.5% of the women in this cohort experienced two or more types of severe or frequent chronic pelvic pain suggests that chronic pelvic pain might well be a substantial health issue in the general population of premenopausal French women.

### 4.2. Strengths and Limitations

Our sample is larger than those of studies that have previously collected data about chronic pelvic pain [3,9,10,11,23,24,25], and our results appear similar to other recent estimates abroad. For example, a long-term cohort study in New Zealand found that at age 38, dysmenorrhea was reported by 46.2% of the women, dyspareunia by 11.6%, and NMCPP by 17.3% whereas in our sample for women aged 35–39 years, it was at 36.7% for dysmenorrhea, 6.7% for dyspareunia and 17.1% for NMCPP [9]. Our sample size ensures enough reliability for estimating prevalence compared to other studies that used a sample about 10 times smaller from ours across other countries (New Zealand, England, Scotland, USA, Denmark) with sample of about 2000 people [3,9,10,11,23,24,25]. Our study was also performed with a sample from the general population of France within different areas across the country which has not previously been used in other works [3,9,10,11,23,24,25]. Questions about women’s health were specially designed for this type of study and we do not have to perform statistical imputation or infer results from other data because of missing results. Moreover, the only requirements for inclusion were health insurance and not medical consultations; the topics were very broad and not limited to pelvic pain even if questions about chronic pelvic pain were specially designed and previously used for this purpose [17,18]. Furthermore, the method used in CONSTANCES to collect data among participants (questionnaires fulfilled during the physical examination within health screening centres) ensures a better reliability in our results than those who send a postal survey to collect their data [23,24,25]. Selection bias seems to be minimal, as shown in our sensitivity analyses, in which reweighting techniques did not change the results for two years of inclusion (2013 and 2014). Our choice not to include women over the age of 49 years old was made to fit our purpose to assess prevalence of chronic pelvic pain among premenopausal women: In the CONSTANCES cohort, the proportion of premenopausal women aged (45–49) were 84% against 38% for those aged (50–54). Moreover, beyond 50-year-olds, some women were taking hormone replacement therapy and stated that they were having periods whereas they do not have to be included in this study.

We might think that women who agreed to be part of the cohort are more likely to present symptoms such as chronic pain because they are offered a free medical examination and health check-up and even a free follow-up. However, this suggests that our estimates for the prevalence of dysmenorrhea, dyspareunia, and NMCPP are reliable and close to the general population of France. Furthermore, the CONSTANCES cohort study, for aspects such as participant selection, was designed to avoid potential selection bias in order to have reliable estimates based on the results drawn from this cohort [13,14,15].

However, a potential assessment bias might be considered for dyspareunia and NMCPP, for which the time period for evaluation of pain symptoms was not specified, whereas assessment of dysmenorrhea was for the last three periods.

### 4.3. Interpretation

Dysmenorrhea appears to be highly prevalent in young women and to decrease with age. This may be partly explained by medication and advice used by women to cope with their pain [26,27]. Another hypothesis is that subsequent pregnancies and deliveries, occurring later, may also reduce dysmenorrhea, which could explain this linear decrease [28]. A similar trend of decreasing rates has been found elsewhere, by the Australian Longitudinal Study of Health and Relationships, where more than 83.8% of the 16- to 19-year-old women but only 65.3% of those aged 40–49 years reported pain during periods [10].

Studies have observed similar trends for dyspareunia, but their rates appear different from ours: 19.4% of young women in Australia compared with 48.8% in this study [10]. This difference may be due in part to the different measures of dyspareunia as it was estimated in this study during the last 12 months. However, the interpretation of rates of dyspareunia should be further studied and interpreted through quality, frequency, and satisfaction of sexual life. As with dysmenorrhea, dyspareunia may decrease with age and the same hypothesis with an improve after subsequent pregnancies are explanations that have been previously reported [9,28]

Also, the prevalence of NMCPP estimated abroad seems to be in the same level than ours with a global prevalence rate of 21.5% in Australia [10] and 20.5% among women of reproductive age in Scotland [25], compared to 17.2% in our sample.

As older women seem to be less concerned by CPP, this might be also explained by the fact that older women are more likely to be further treated for these pains, explaining this decrease. However, the counterpart is that our estimates might be underestimated because we did not assess treatments of CPP.

However, each symptom of chronic pelvic pain seems to be on the high side compared to other studies abroad [3,9,10,11]. This emphasizes the fact that it is likely an underestimated symptom and a burden that women may carry during their menstrual life. Practitioners should be aware of this chronic condition.

Severe dysmenorrhea, non-menstrual pelvic pain, and deep dyspareunia affecting daily activities or sexual life are all symptoms that suggest endometriosis, which has a diagnostic delay estimated at 7 to 10 years in some European countries [18,29,30,31]. It is known that severe dysmenorrhea is one of the symptoms associated with severe endometriosis as a deep infiltrating disease [17,32]. One study of 1000 women with endometriosis reported that 70% of them stated that they had at least two of the three types of pelvic pain [33]. If we consider that numerous combinations of at least two of severe dysmenorrhea, often or always dyspareunia, and NMCPP, might be symptoms of endometriosis, it means that these women should be referred to specialized medical centres for diagnosis and treatment. Thus, women, and especially the youngest or those for whom multiple severe or frequent symptoms impair their daily activities and negatively affect their academic performance, should be offered medical assistance [34]. The aim of this medical support would be to improve these symptoms and their consequences (absence from work, psychological distress, sexual dysfunction, infertility, and increased medical expenses) knowing that only a few reported seeing a doctor for period pain [34,35]. Then, this specific proportion of women (7.5%) experiencing severe and/or frequent symptoms should be further analyzed to assess potential characteristics and factors associated that may help in caring for this peculiar proportion of women.

Our conclusion is that chronic pelvic pain among women is an underestimated and neglected condition and health practitioners should be aware of this in order to improve the medical assistance and support that should be proposed. A substantial proportion of French women experience multiple severe or frequent pelvic pain symptoms which are elements that may suggest diseases such as endometriosis: severe dysmenorrhea, frequent dyspareunia, and non-menstrual chronic pelvic pain. These women should be further analyzed to have a better characterization of their symptoms and the consequences on their daily life (impact on sexual life or on work efficiency) as they are probably neglected by medical staff. More attention should be paid to these women of reproductive age because they might be proposed for medical assistance and specific follow-up.

## Figures and Tables

**Table 1:** Comparison of baseline characteristics among participants, *n* = 21,287.

Age of Participants Number of Participants (Range, y)	(18–24) *n* = 2386 *n* (%)	(25–29) *n* = 3493 *n* (%)	(30–34) *n* = 3821 *n* (%)	(35–39) *n* = 4052 *n* (%)	(40–44) *n* = 4180 *n* (%)	(45–49) *n* = 3355 *n* (%)	Total *n* = 21,287 *n* (%)
**Body Mass Index (BMI), (kg/m²)**							
BMI < 18.5 (*n* = 1133)	223 (9.5)	198 (5.8)	235 (6.3)	223 (5.7)	155 (3.8)	99 (3.0)	1133(5.5)
18.5 < BMI < 25 (*n* = 14,039)	1686 (72.2)	2448 (72.0)	2586 (69.7)	2622 (66.6)	2628 (64.6)	2069 (63.3)	14,039 (67.8)
25 < BMI < 30 (*n* = 3778)	311 (13.3)	510 (15.0)	633 (17.1)	728 (18.5)	844 (20.8)	752 (23.0)	3778 (18.2)
30 < BMI < 35 (*n* = 1205)	84 (3.6)	162 (4.8)	187 (5.0)	230 (5.8)	298 (7.3)	244 (7.5)	1205 (5.8)
35 < BMI < 40 (*n* = 408)	25 (1.1)	60 (1.8)	50 (1.3)	92 (2.3)	102 (2.5)	79 (2.4)	408 (2.0)
BMI > 40 (*n* = 153)	7 (0.3)	21 (0.6)	21 (0.6)	41 (1.0)	38 (0.9)	25 (0.8)	153 (0.7)
**Smoking status**							
Never smoker (*n* = 10,312)	1313 (56.4)	1846 (54.4)	1701 (46.0)	1936 (49.3)	2020 (50.1)	1496 (46.3)	10,312 (50.1)
Former smoker (*n* = 5226)	262 (11.3)	573 (16.9)	952 (25.8)	1123 (28.6)	1181 (29.3)	1135 (35.2)	5226 (25.4)
Smoker (*n* = 5062)	752 (32.3)	976 (28.7)	1042 (28.2)	866 (22.1)	828 (20.6)	598 (18.5)	5062 (24.6)
**Employment status**							
Not working (*n* = 3624)	1280 (54.7)	618 (18.0)	488 (13.0)	480 (12.1)	435 (10.6)	323 (9.8)	3624 (17.3)
Working (*n* = 17,311)	1060 (45.3)	2817 (82.0)	3272 (87.0)	3499 (87.9)	3685 (89.4)	2978 (90.2)	17311(82.7)
**Geographic origin**							
Mainland France (*n* = 18,915)	2190 (92.2)	3151 (90.6)	3357 (88.2)	3511 (87.0)	3709 (89.1)	2997 (89.7)	18,915 (89.2)
French overseas territories (*n* = 252)	49 (2.1)	52 (1.5)	51 (1.3)	44 (1.1)	32 (0.8)	24 (0.7)	252 (1.2)
Europe (*n* = 909)	58 (2.4)	112 (3.2)	173 (4.5)	200 (5.0)	197 (4.7)	169 (5.1)	909 (4.3)
Africa (*n* = 588)	43 (1.8)	96 (2.8)	109 (2.9)	141 (3.5)	126 (3.0)	73 (2.2)	588 (2.8)
Asia (*n* = 250)	18 (0.8)	30 (0.9)	53 (1.4)	69 (1.7)	49 (1.2)	31 (0.9)	250 (1.2)
Other (*n* = 286)	18 (0.8)	36 (1.0)	65 (1.7)	70 (1.7)	50 (1.2)	47 (1.4)	286 (1.3)
**Duration of periods (days)**							
(0–4) (*n* = 9146)	2088 (88.7)	3042 (88.1)	3290 (87.0)	3508 (87.8)	3589 (86.7)	2552 (77.2)	9146 (43.2)
(5–15) (*n* = 12,014)	265 (11.3)	411 (11.9)	491 (13.0)	488 (12.2)	551 (13.3)	755 (22.8)	12,014 (56.8)
**Parity**							
P0 (*n* = 8958)	2252 (98.0)	2813 (82.7)	1788 (47.8)	936 (23.8)	695 (17.1)	474 (14.7)	8958 (43.3)
P1 (*n* = 3309)	41 (1.8)	376 (11.0)	852 (22.8)	724 (18.4)	750 (18.4)	566 (17.5)	3309 (16.0)
P2 (*n* = 5816)	4 (0.2)	179 (5.3)	895 (23.9)	1667 (42.4)	1743 (42.9)	1328 (41.1)	5816 (28.1)
P3+ (*n* = 2594)	0 (0.0)	35 (1.0)	204 (5.5)	609 (15.5)	879 (21.6)	867 (26.8)	2594 (12.5)
**Contraception method**							
None (*n* = 5395)	280 (12.2)	702 (20.7)	988 (26.5)	1050 (26.6)	1274 (31.9)	1101 (34.6)	5395 (26.3)
Mechanic (condom, cup) (*n* = 3425)	324 (14.1)	532 (15.7)	703 (18.9)	736 (18.6)	638 (16.0)	492 (15.5)	3425 (16.7)
Sterilization (*n* = 471)	0 (0.0)	1 (0.0)	5 (0.1)	48 (1.2)	193 (4.8)	224 (7.0)	471 (2.3)
Copper intrauterine device (*n* = 3246)	122 (5.3)	362 (10.7)	595 (16.0)	849 (21.5)	737 (18.5)	581 (18.3)	3246 (15.8)
Hormonal intrauterine device (*n* = 1169)	18 (0.8)	56 (1.7)	157 (4.2)	306 (7.8)	338 (8.5)	294 (9.3)	1169 (5.7)
Hormonal (pills, implant...) (*n* = 6816)	1550 (67.6)	1737 (51.2)	1276 (34.3)	958 (24.3)	809 (20.3)	486 (15.3)	6816 (33.2)

## Supplementary Materials

The following are available online at https://www.mdpi.com/article/10.3390/jcm10112481/s1. Figure S1. Summary of the difference between the estimate of prevalence without and with weighting within each of the three components of chronic pelvic pain (CPP) (none, mild moderate and severe dysmenorrhea none, sometimes often or always dyspareunia, non-menstrual CPP (NMCPP)) for each age group. *n* = 6191; Figure S2. Distribution of the effects of the weighting within estimations of components of CPP according to level of each prevalence estimates (none, mild moderate and severe dysmenorrhea none, sometimes often or always dyspareunia NMCPP) for each age group. *n* = 6191.
