# Supplementary material for: The Underestimated Prevalence of Neglected Chronic Pelvic Pain in Women, a Nationwide Cross-Sectional Study in France
Source: J Clin Med. 2021 Jun 3;10(11):2481. doi: 10.3390/jcm10112481 (PMC8199870; doi:10.3390/jcm10112481)
Supplement: Supplementary file 1 [file jcm-10-02481-s001.zip › jcm-1207833-supplementary.pdf]

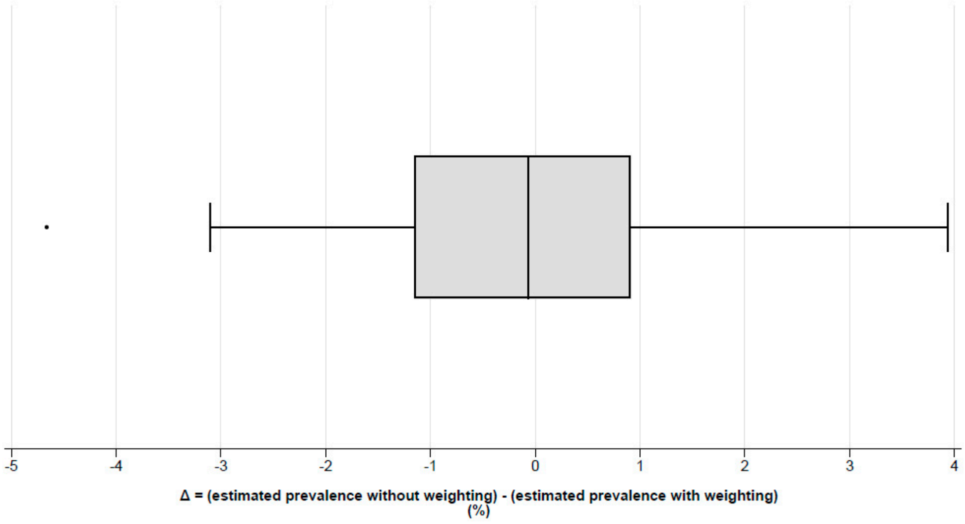

Figure S1: Summary of the difference between the estimate of prevalence without and with weighting within each of the three components of CPP (none, mild moderate and severe dysmenorrhea / none, sometimes often or always dyspareunia / NMCPP) for each age group. N = 6191.

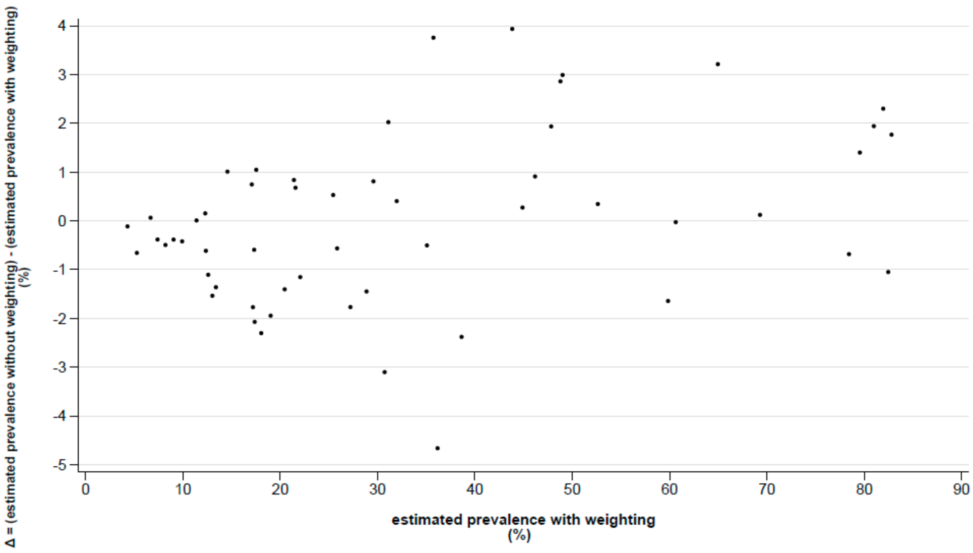

Figure S2: Distribution of the effects of the weighting within estimations of components of CPP according to level of each prevalence estimates (none, mild moderate and severe dysmenorrhea / none, sometimes often or always dyspareunia / NMCPP) for each age group. N = 6191.
